# An integrative review of leadership competencies and attributes in advanced nursing practice

**DOI:** 10.1111/jan.14092

**Published:** 2019-07-21

**Authors:** Maud Heinen, Catharina van Oostveen, Jeroen Peters, Hester Vermeulen, Anita Huis

**Affiliations:** ^1^ Radboud University Medical Center, Radboud Institute for Health Sciences Scientific Institute for Quality of Healthcare Nijmegen The Netherlands; ^2^ Spaarne Gasthuis Hospital, Spaarne Gasthuis Academy Haarlem The Netherlands; ^3^ Erasmus School of Health Policy & Management Erasmus University Rotterdam Rotterdam The Netherlands; ^4^ Hogeschool van Arnhem en Nijmegen HAN University of Applied Sciences Nijmegen The Netherlands; ^5^ HAN University of Applied Sciences Nijmegen The Netherlands

**Keywords:** advanced nursing practice, clinical nurse leaders, competency, education, leadership, literature review

## Abstract

**Aim:**

To establish what leadership competencies are expected of master level‐educated nurses like the Advanced Practice Nurses and the Clinical Nurse Leaders as described in the international literature.

**Background:**

Developments in health care ask for well‐trained nurse leaders. Advanced Practice Nurses and Clinical Nurse Leaders are ideally positioned to lead healthcare reform in nursing. Nurses should be adequately equipped for this role based on internationally defined leadership competencies. Therefore, identifying leadership competencies and related attributes internationally is needed.

**Design:**

Integrative review.

**Methods:**

Embase, Medline and CINAHL databases were searched (January 2005–December 2018). Also, websites of international professional nursing organizations were searched for frameworks on leadership competencies. Study and framework selection, identification of competencies, quality appraisal of included studies and analysis of data were independently conducted by two researchers.

**Results:**

Fifteen studies and seven competency frameworks were included. Synthesis of 150 identified competencies led to a set of 30 core competencies in the clinical, professional, health systems. and health policy leadership domains. Most competencies fitted in one single domain the health policy domain contained the least competencies.

**Conclusions:**

This synthesis of 30 core competencies within four leadership domains can be used for further development of evidence‐based curricula on leadership. Next steps include further refining of competencies, addressing gaps, and the linking of knowledge, skills, and attributes.

**Impact:**

These findings contribute to leadership development for Advanced Practice Nurses and Clinical Nurse Leaders while aiming at improved health service delivery and guiding of health policies and reforms.

## 1 INTRODUCTION

Developments in health care, like a growing number of patients with chronic diseases, an increased complexity of patients, a stronger focus on person‐centred care and a demand for less institutionalized care ask for well‐trained master level‐educated nurses operating as partners in integrated care teams, with leadership qualities at all levels of the healthcare system. Changes in health care are also underlined by a definition of health as proposed by Huber et al. (Huber et al., 2011) where health is defined as ‘the ability to adapt and self manage in the face of social, physical and emotional challenges’ as a refinement of the World Health Organization (WHO) definition where health is ‘a state of complete physical, mental and social well being’ (WHO, 1948). This stipulates the de‐medicalization of health care and society and emphasizes the need for change in the way health care is organized. Also the Institute of Medicine with their report on ‘The Future of Nursing’ supports the urge for nurses to take their roles to address changes in health care (IOM, 2011). However leading change is a complex and not yet well understood process (Nelson‐Brantley & Ford, 2017). Therefore, especially master level‐educated nurses have to be trained in leadership based on internationally established leadership competencies. This review investigates what leadership competencies are expected from and can be identified for master educated nurses from an international perspective.

### 1.1 Background

Clinical nurses who are trained at master's level, for example, Advanced Practice Nurses (APNs) and Clinical Nurse Leaders (CNLs), are in a unique position to take a leadership role, in collaboration with other healthcare professionals, to shape healthcare reform, as they use extended and expanded skills and are trained to focus on improved patient outcomes, the application of evidence‐based practice and assessing cost‐effectiveness of care (Stanley et al., 2008). The focus of this review is on APNs and CNLs, where APN is regarded as a general designation for all nurses with an advanced degree in a nursing program, that is, Certified Nurse Practitioner (NP), Certified Registered Nurse Anaesthetist, Certified Nurse Midwife and Clinical Nurse Specialist (CNS) (APRN Joint Dialogue Group, 2008)*.* APNs are prepared with specialized education in a defined clinical area of practice. With APN in this review, we refer to the NP and the CNS. The CNL is educated to improve the quality of care and coordinate care in general through collaboration at the microsystems level in the entire healthcare team (APRN Joint Dialogue Group, 2007). Both groups of professionals are trained to integrate science in practice and education, have increased degrees of autonomy in judgments and clinical interventions and are expected to be engaged in collaborative and inter professional practices to achieve the best outcomes for patients, personnel and organization (American Association of Colleges of Nursing, 2011). They are also expected to substantially contribute to clinical outcomes through, that is, continuous quality improvement in patient care and creating a supportive environment for their colleagues, and to contribute to the development of their profession, healthcare systems and healthcare policy. (American Association of Colleges of Nursing, 2004; Bender, Williams, & Su, 2016; Hamric, Hanson, Tracy, & O'Grady, 2014). Therefore developing leadership competencies is an essential prerequisite for these master educated nurses, APNs however appear to experience a lot of difficulties in enacting their leadership role (Begley, Murphy, Higgins, & Cooney, 2014; Elliott, Begley, Sheaf, & Higgins, 2016a).

Leadership is subject of many discussions can be regarded from different perspectives and is mostly related to specific contexts. Hence, there is no single definition applicable to all settings and professions. Leadership is mostly regarded in relation to managing a team or organization (Gosling & Mintzberg, 2003) but can also be defined as a set of personal skills or traits, or focussing on the relation between leaders and followers (Alimo‐Metcalfe & Alban‐Metcalfe, 2004; Bolden, 2004). Transformational and situational leadership are also commonly used concepts where transformational leadership is regarded as the process of leading and inspiring a group to achieve a common goal (Northouse, 2014) and situational leadership is focusing on the interaction between individual leadership styles and the features of the environment or situation where the leader is operating. (Fiedler, 1967; Hamric et al., 2014; Lynch, McCormack, & McCance, 2011). In this review, leadership is regarded as a process where nurses can develop observable leadership competencies and attributes needed to improve patient outcomes, and personnel and organizational outcomes (Kouzes & Posner, 2012). This implies that leadership competencies can be viewed as intended and defined outcomes of learning and that leadership and leadership competencies are not restricted to one single theory. A competency can be defined as ‘an expected level of performance that results from an integration of knowledge, skills, abilities and judgment’ (American Nurses Association, 2013).

The lack of an unambiguous definition of leadership in clinical practice, including clearly defined leadership competencies in nursing, is reflected in education. For most training programs and curricula, it is unclear whether the profiles used in education are up‐to‐date and aiming` at internationally accepted leadership competencies with evidence‐based methods to achieve these competencies. To enhance leadership qualities in master educated nurses, it is necessary to explicitly define what leadership competencies are expected from APNs and CNLs (Delamaire & Lafortune, 2010). Identifying and establishing internationally agreed on leadership competencies in master educated nurses is a first step to developing evidence‐based curricula on leadership (Falk‐Rafael, 2005; Vance & Larson, 2002). Such a curriculum facilitates APN and CNL students to not only become competent clinical and professional leaders but also well‐prepared for organizational systems and political leadership (Hamric et al., 2014). As such, it enables them to have a positive and significant impact on patient, personnel and organizational level outcomes. Accordingly, this review aims to identify and integrate leadership competencies of the master level‐educated nurse (APN and CNL) from an international perspective.

## 2 THE REVIEW

Based on the decision flowchart developed by Flemming et al. (Flemming, Booth, Hannes, Cargo, & Noyes, 2018), this review was reported according to the Preferred Reporting Items for Systematic Reviews and Meta‐Analyses statement (Moher, Liberati, Tetzlaff, & Altman, 2009) and the Enhancing transparency in reporting the synthesis of qualitative research statement (Tong, Flemming, McInnes, Oliver, & Craig, 2012).

### 2.1 Aim

To identify and integrate leadership competencies of the master level‐educated nurse (APN and CNL) from an international perspective.

### 2.2 Design

An integrative review design was used, which allows for the combination of various study designs and data sources to be included. In using this methodology, a rigorous and systematic approach is ensured (Whittemore & Knafl, 2005). We followed the five stage methodology by Whittemore and Knafl (Whittemore & Knafl, 2005), however for the data synthesis phase, we used the four leadership domains of Hamric et al (Hamric et al., 2014; Hamric, Spross, & Hanson, 2009) as an a priori framework to integrate the extracted data.

The APN Leadership competency is conceptualized by Hamric et al. (Hamric et al., 2014) as occurring in four primary domains; in clinical practice with patients and staff, in professional organizations, in healthcare systems and in health policy‐making arenas. As stated above, this review focuses on the leadership competencies of APNs and CNLs. Additionally, knowledge, skills and attributes (KSA) needed to develop leadership competencies were topic of interest, where knowledge is regarded as being acquired through cognitive learning, skills through practice and attributes as behaviours that are learned over time (Koolen, 2016). We would like to add a reference to support this one, the full reference is added to the remark concerning Koolen in the reference list. The reference that needs to be added here is; ​Guillén and Saris (2013)

### 2.3 Search methods

First, MEDLINE, EMBASE and CINAHL databases were searched from January 2005 ‐ December 2018 to identify articles concerning leadership in APNs and CNLs. To find all literature fitting our scope, we used the words attitude* role* attribute* next to leadership and competenc*. The search strategy was designed and conducted with the help of a clinical librarian (Data S1).

Articles were eligible if they explicitly described leadership competencies or related knowledge, skills or attributes in: (a) studies reporting on theory or theoretical leadership models; (b) developmental studies on leadership programmes (c) studies reporting on the effects of leadership programmes. No restrictions on study designs were applied. Studies were excluded when they concerned managerial leadership, if they did not concern APNs or CNLs (i.e., bachelor nurses and/or undergraduate nurses); or described leadership styles in general. Box gives an overview of in and exclusion criteria.

BOX 1 **Inclusion and exclusion criteria.**

1

**Table:** 

Inclusion	Exclusion
January 2005–December 2018 Research Studies on the development of theory/ theoretical models concerning leadership (APN and CNL) describing leadership competencies Studies on the development of leadership programmes (APN and CNL) describing leadership competencies Studies on effectiveness of leadership programmes (APN and CNL) describing leadership competencies Studies concerning o ‘Clinical leadership’ o ‘Professional leadership’ o ‘Systems leadership’ o ‘Health Policy leadership’ o Settings: Acute care, Long term care, Home care, Mental care o Education APN and CNL Location: Europe, North America, Australia	Editorials, opinion papers, interviews Studies concerning effectiveness of leadership on nurses turnover and patient outcomes (transformational leadership) Studies concerning the effects of leadership on the quality of care or quality improvement Specific leadership styles, e.g. hierarchical leadership, transformational leadership etc. Management leadership Managers of nursing wards Governance South American, Asian and African countries, due to expected large cultural differences with regard to leadership in nursing.

Secondly, the websites of international professional nursing organizations were searched for documents on leadership competencies in NPs, CNSs, and CNLs. Worldwide, there are more than 100 nursing organizations, usually part of one umbrella association or council. Therefore, this review focused on frameworks of umbrella organizations in Australia, Europe, and North America and international nursing councils. Frameworks had to describe nursing leadership and related competencies in NPs, CNSs, or CNLs.

Eligible articles and frameworks were independently selected by three reviewers (MH, AH, CvO) based on the relevance of their titles and abstracts, as retrieved by the search. If articles met the inclusion criteria, full‐text versions of the articles were obtained and further scrutinized for eligibility by (MH, AH, CvO). HV was involved in any cases of disagreement, where consensus was reached through discussion. The reference lists of included articles were checked to detect any potential additional studies.

### 2.4 Search outcome

**Figure 1:**
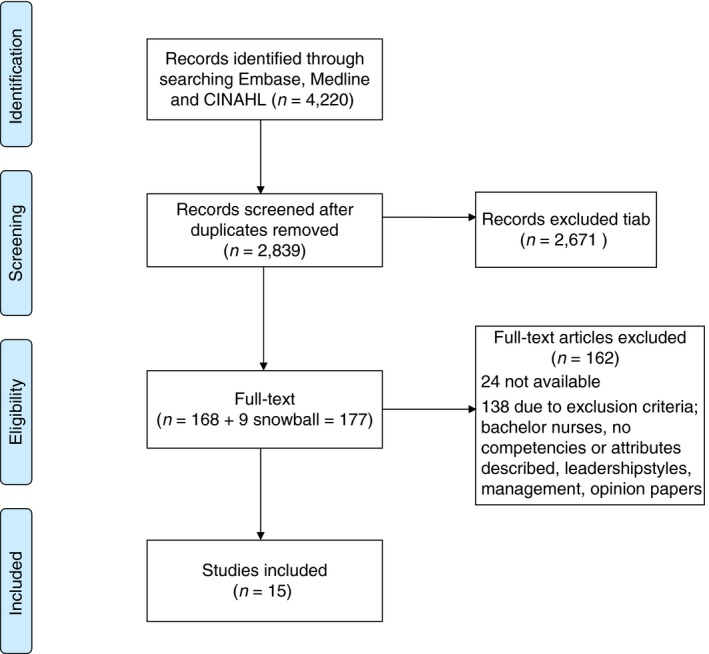
Flow diagram (PRISMA 2009) [Colour figure can be viewed at http://www.wileyonlinelibrary.com/]

The search strategy in PUBMED, CINAHL, and EMBASE resulted initially in 4,220 records. After removing duplicates, the remaining 2,839 articles were screened on title and abstract. As a result, 168 articles and nine additional articles, added through reference checking, were included for full‐text assessment. Twenty‐four articles were not available in full text. Fifteen articles were eventually included in this review. The flow diagram (Figure 1) gives an overview of the inclusion process.

### 2.5 Quality appraisal

A quality appraisal (Data S2) was conducted by two researchers (MH, AH) on all 15 studies. Quality appraisal of the included studies was conducted using the Mixed methods Appraisal Tool MMAT (Hong, Gonzalez‐Reyes, & Pluye, 2018). The MMAT is a critical appraisal tool that is designed for the appraisal stage of systematic mixed studies reviews. It permits to appraise the methodological quality of five categories studies. The MMAT starts with two screening questions to determine whether the study is an empirical study and the tool can be used. For each category, five criteria are defined to rate the quality of the studies. It is advised not to calculate an overall score from the ratings of each criterion and excluding studies with low methodological quality is discouraged. Quality was therefore not used to include or exclude studies from the review, also because of the difficulties in comparing quality of studies using different designs (Whittemore & Knafl, 2005). The goal of the quality appraisal was to evaluate the quality of studies and the degree of evidence in an unbiased and transparent way. A quality appraisal of included frameworks was not conducted.

### 2.6 Data extraction

Data extraction was performed using a pre‐defined, structured data extraction sheet and was double‐checked by three researchers (MH, AH, CvO). The following data were extracted: author, year of publication, title, methodology, country and setting, master's APNs or CNLs. Competencies and KSA were derived from the frameworks and studies, by the same three researchers (MH, AH, CvO). Involvement of three independent researchers was used to ensure rigour of data extraction (Whittemore & Knafl, 2005).

### 2.7 Synthesis

Competencies described in the original studies subsequently were designated to the leadership domains described by Hamric et al. (Hamric et al., 2014) by three researchers (MH, AH, CvO). In cases of discrepancy, the selected domains were discussed until consensus was reached. The next step consisted of clustering of overlapping competencies by two researchers (MH, AH), which were checked by a third researcher (CvO). The competency from the overlapping items that best described the content was chosen for the final overview of competencies, sometimes with a minor adaptation to fully grasp the essence of this competency. The same process was followed for the KSA‐items.

## 3 RESULTS

### 3.1 Individual studies

One out of 15 articles concerned both the NP and the CNS, seven were about the NP, three were about the CNS and four articles focused on the CNL. Most articles (9/15) originated from the United States of America (USA), three from Australia and three articles originated from Canada, the UK, and Finland respectively. Two articles published different aspects of the same research (Carryer, Gardner, Dunn, & Gardner, 2007; Gardner, Carryer, Gardner, & Dunn, 2006) (Table 1).

**Table 1:** Overview of included studies (15) and frameworks (7)

First Author/ Organization	Year	Title	Methodology and aim study/ Short description framework	Participants	Country	NP/CNS/ CNL
Studies						
1. Ailey	2015	Educating nursing students in clinical leadership	Case study/ To describe the use of Situated Learning in Nursing Leadership in CNL education	22 Generalist master students	USA	CNL
2. Bahouth	2011	Centralized resources for nurse practitioners: common early experiences among leaders of six large health systems	Survey and focus group discussions/ To describe experiences of implementing a leadership role for hospital‐based NPs	6 Leaders of academic institutions	USA	NP
3. Bearnholdt	2011	The Clinical Nurse Leader – new nursing role with global implications	Short report of the literature – CNL role and education development	NA	USA	CNL
4. Bender	2016	Refining and validating a conceptual model of Clinical Nurse Leader integrated care delivery	Sequential mixed methods combining initial qualitative (model refinement and survey development) and subsequent quantitative (survey) administration and analysis) approaches/ To empirical validate a conceptual model of CNL integrated care delivery	CNLs, clinicians, administrators involved in CNL initiatives	USA	CNS
5. Carryer	2007	The core role of the nurse practitioner: Practice, professionalism and clinical leadership	Interviews/ To draw on empirical evidence to illustrate the core role of nurse practitioners	15 Nurse practitioners	New Zealand & Australia	NP
6. Gardner	2006	Nurse practitioner competency standards: findings from collaborative Australian and New Zealand research	Interpretive synthesis with multiple data sources published data of policies and curricula/ To develop core standards that could inform nurse practitioner competencies	NA	Australia & New Zealand	NP
7. Gerard	2012	Course strategies for clinical nurse leader development	Description and qualitative evaluation of course strategies for clinical nurse leader development	9 Nursing master students	USA	CNL
8. Goldberg	2016	Development of a curriculum for advanced nurse practitioners working with older people with frailty in the acute hospital through a modified Delphi process	Literature review, workshops and a three round modified Delphi‐study/ To establish an expert consensus on the role description and essential competencies for ANPs	31 experts	UK	NP
9. Leggat	2015	Developing clinical leaders: the impact of an action learning mentoring programme for advanced practice nurses	Pre‐post longitudinal intervention study/ To determine whether a formal mentoring programme assists nurse practitioner candidates to develop competence in the clinical leadership competencies	18 NP candidates, 17 senior nurses	Australia	NP
10. Maag	2006	A Conceptual Framework for a Clinical Nurse Leader Program	Description of and explaining the components of the conceptual model for a CNL educational program	NA	USA	CNL
11. Nieminen	2011	Advanced practice nurses' scope of practice: a qualitative study of advanced clinical competencies	Qualitative/ To describe and explore Advanced Practice Nurses’ clinical competencies and how these are expressed in clinical practice	26 APN and 6 APN students	Finland	NP
12. Kalb	2006	A competency‐based approach to public health nursing performance appraisal	Pilot testing of assessment tool, developed based on a review of public health nurse competency literature/ To integrate public health nursing competencies into a comprehensive review instrument	50 Nurses from PHN workforce	USA	NP/ CNS
13. O'Rourke	2016	Activities and Attributes of Nurse Practitioner Leaders: Lessons from a Primary Care System Change	Interviews and document analysis/ To examine the activities and attributes of two NP leaders	6 Healthcare providers, 3 managers and 7 health policy advisors	Canada	NP
14. Thompson	2011	Clinical Nurse Specialist Education; Actualizing the Systems Leadership competency	Overview of educational strategies aiding in the acquisition of systems leadership and change agent skills of CNS/ To show how sequenced educational strategies aid in the acquisition of systems leadership and change agent skills	NA	USA	CNS
15. Sievers	2006	Achieving Clinical Nurse specialist Competencies and Outcomes Through Interdisciplinary Education	Plan do study act cycles/ To create an interdisciplinary educational experience for clinical nurse specialist (CNS) students	7 Learners	USA	CNS
Frameworks						
1. American Association of Colleges of Nursing	2013	Master's Essentials and Clinical Nurse Leader® Competencies	The Master's Essentials & Clinical Nurse Leader Competencies are imbedded in 9 domains. Core leadership competencies are mainly described in the essential ‘Organizational and Systems Leadership’	NA	USA	CNL
2. American Association of Colleges of Nursing	2006	The Essentials of Doctoral Education for Advanced Nursing Practice,	Leadership competencies and roles are imbedded in eight domains	NA	USA	NP
3. ANMC	2014	Nurse practitioner standards for practice	The leadership domain is couched within the clinically focused standards.	NA	Australia	NP
4. The Canadian Nurses Association	2010	Canadian nurse practitioner core competency framework	Leadership competencies within the category ‘Professional Role, Responsibility and Accountability’	NA	Canada	NP
5. ICN	2015	International Council of Nurses Leadership For Change™ (LFC) program	Leadership competencies & roles are focused on 3 strategic aims and include 11 defined outcomes	NA	Europe	CNL
6. The National Organization of Clinical Nurse Specialists	2008	Clinical Nurse Specialist Core Competencies	System Leadership competency is one of the 7 Clinical Nurse Specialist core competencies, described by behaviour, sphere of influence and nurse characteristics needed.	NA	USA	CNS
7. The National Organization of Nurse Practotioner Faculties	2014	A delineation of suggested content specific to the NP core competencies,	Leadership is 1 of 9 domains, the leadership domain itself includes 7 competencies	NA	USA	NP

Abbreviation: NA, Not Applicable.

Sample sizes were relatively small, ranging from 6‐50 respondents and consisted of nurse leaders (Bahouth et al., 2013; Goldberg et al., 2016; O'Rourke & Higuchi, 2016), experienced nurses (Bender, Williams, Su, & Hites, 2017; Carryer et al., 2007; Gardner et al., 2006; Kalb et al., 2006; Leggat, Balding, & Schiftan, 2015; Nieminen, Mannevaara, & Fagerström, 2011) and APN or CNL students (Ailey, Lamb, Friese, & Christopher, 2015; Gerard, Grossman, & Godfrey, 2012; Leggat et al., 2015; Nieminen et al., 2011; Sievers & Wolf, 2006).

Multiple research designs were used. These included surveys, interviews, and focus groups to describe experiences on integrating NPs and CNSs into hospitals (Bahouth et al., 2013; O'Rourke & Higuchi, 2016; Sievers & Wolf, 2006) and expressed clinical competences (Nieminen et al., 2011), a case study on an education program for CNLs (Ailey et al., 2015), exploring the effect of a mentor program of NP students on developing leadership competencies (Leggat et al., 2015), piloting an assessment for performance review of NPs and CNSs (Kalb et al., 2006) and multi‐method research to develop shared competencies and educational standards for APNs (Bender et al., 2017; Carryer et al., 2007; Gardner et al., 2006; Goldberg et al., 2016). Eight were descriptive studies on (experiences with) educational programs for CNLs or CNSs (Ailey et al., 2015; Baernholdt & Cottingham, 2011; Gerard et al., 2012; Goldberg et al., 2016; Leggat et al., 2015; Maag, Buccheri, Capella, & Jennings, 2006; Sievers & Wolf, 2006; Thompson & Nelson‐Marten, 2011) Baernholdt and Cottingham (Baernholdt & Cottingham, 2011) also reported on the development of the CNL role in practice. Six studies explicitly described leadership competencies (Bender et al., 2017; Gardner et al., 2006; Gerard et al., 2012; Goldberg et al., 2016; Kalb et al., 2006; Nieminen et al., 2011). Furthermore, studies focused on knowledge (Ailey et al., 2015; Carryer et al., 2007), leadership skills (Baernholdt & Cottingham, 2011; Maag et al., 2006; Thompson & Nelson‐Marten, 2011) and leadership attributes (Bahouth et al., 2013; Sievers & Wolf, 2006).

For eight out of 15 studies, quality could not be determined on the basis of quality appraisal tools for research (Data S2), five studies scored positive on all five MMET domains (Bender et al., 2017; Carryer et al., 2007; Goldberg et al., 2016; Nieminen et al., 2011; O'Rourke & Higuchi, 2016), one study scored positive on four out of five domains (Leggat et al., 2015) and one study scored positive on one domain (Bahouth et al., 2013).

### 3.2 Frameworks

Seven competency frameworks, including leadership competencies, were identified. The frameworks were developed between 2006 and 2014 and originated internationally in Europe (1/7) (ICN, 2015), the USA (4/7) (American Association of Colleges of Nursing, 2006, 2013; The National Organization of Nurse Practotioner Faculties, 2014), Canada (1/7) (The Canadian Nurses Association, 2010) and Australia (1/7) (Nursing and Midwifery Board of Australia, 2014). All frameworks describe leadership competencies for the NP, CNS, or CNL but the extent to which the four leadership domains (i.e., clinical‐, professional‐, system‐, and health policy leadership) are covered differed (Table 1). In Australia, leadership is linked to four defined practice standards in the nursing process. Additionally, leadership is defined as the ability to lead care teams where the NP supports other professionals through clinical supervision and mentoring (Nursing and Midwifery Board of Australia, 2014). The Canadian Nurse Practitioner Core Competencies Framework identifies leadership as a core competence for the NP that should be reflected in excellent clinical practice and by mentoring colleagues and students. Leadership activities should not be limited to the NPs' own practice or institution but should focus on the entire care continuum, also including the political field of health care (The Canadian Nurses Association, 2010). The NONPF‐USA defines nursing leadership as the ability to change care systems, create partnerships, establish adequate communication and to participate in professional organizations (The National Organization of Nurse Practotioner Faculties, 2014). The Clinical Nurse Specialist Core Competencies Framework has assigned leadership competencies mainly to the heading ‘System leadership’ and describes specific leadership behaviour and associated sphere of influence and nurse characteristics needed (The National Organization of Clinical Nurse Specialists, 2010). The Essentials of Doctoral Education for Advanced Nursing Practice (American Association of Colleges of Nursing, 2006) is designed to prepare nurses for the highest level of leadership in practice and scientific inquiry.

Leadership competencies mainly refer to the category ‘Organizational and system leadership for quality improvement and systems thinking’. Leadership competencies are applied in clinical practice, as well in the entire field of health care. The ‘Master's Essentials and Clinical Nurse Leader Competencies’ outlined in the ‘Competencies and Curricular Expectations for Clinical Nurse Leader Education and Practice’ (American Association of Colleges of Nursing, 2013) describes the CNL as ‘a leader in the healthcare delivery system in all settings where healthcare is delivered’ (American Association of Colleges of Nursing, 2013, p. 4). The leader competencies are embedded in nine categories, with the core leadership competencies mainly described in ‘Essential 2: Organisational and Systems Leadership’. Finally, the International Council of Nurses Leadership for Change™ (LFC) program is developed to prepare nurses to take a leadership role during health sector change and reform and enhance their contribution to health services (ICN, 2015). Leadership competencies are mainly focused on a system‐ and health policy leadership. Four frameworks provide suggestions for curriculum development concerning required KSA or performance indicators (ICN, 2015).

### 3.3 Data synthesis

**Figure 2:**
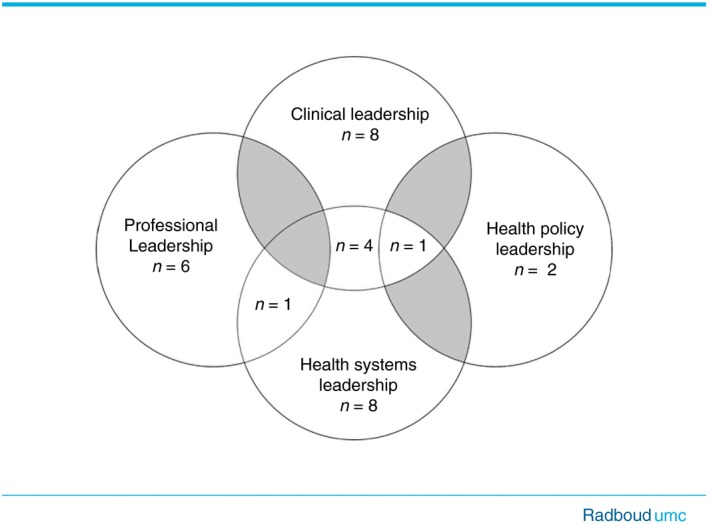
Model competencies [Colour figure can be viewed at http://www.wileyonlinelibrary.com/]

The 150 competencies derived from the literature are displayed in Data S3. Table 2 shows the final synthesis of the extracted competencies which resulted in the identification of 30 core leadership competencies, assigned to the four leadership domains of Hamric et al. (Hamric et al., 2014). The highest number of competencies (*n* = 8) was designated to the clinical and to the systems leadership domains, six to the professional and two to the health policy leadership domains. Six competencies fitted more than one domain, of which one competency related to three domains, the clinical, the health systems, and the health policy domains and four competencies were linked to the clinical, and to the health systems leadership domains. One competency was designated to the professional and the health systems leadership domains. The model in Figure 2 presents this synthesis of competencies.

**Table 2:** Final 30 leadership Core competencies within (four) leadership domains

Clinical Leadership domain – Core competencies (*N* = 8) Provides leadership to the healthcare team to promote health, facilitate self‐care management, optimize patient engagement, and prevent future decline including progression to higher levels of care and readmissions. Acts as a resource person, preceptor, mentor/coach, and role model demonstrating critical and reflective thinking Assumes as a clinical expert, a leadership role in establishing and monitoring standards of practice to improve client care, including intra‐ and interdisciplinary peer supervision and review Analyses organizational systems for barriers and promotes enhancements that affect client healthcare status. Engages in advanced nursing practice and provide leadership for evidence‐based practice. This requires competence in knowledge application activities: identifies current relevant scientific health information, the translation of research in practice, the evaluation of practice, improvement of the reliability of healthcare practice and outcomes, and participation in collaborative research Provides leadership and acts as a liaison with other health agencies and professionals, and participates in assessing and evaluating healthcare services to optimize outcomes for patients/clients/communities Collaborates with healthcare professionals, including physicians, advanced practice nurses, nurse managers, and others, to plan, implement, and evaluate an improvement opportunity. Aligns practice with overall organizational/ contextual goals Guides, initiates, and provides leadership in 1) the development and implementation of standards, practice guidelines, quality assurance, and 2) education, and 3) research initiatives.
Professional Leadership domain – Core competencies (*N* = 6) Participates in professional organizations and activities that influence advanced practice nursing Provides leadership in the development and integration of the nurse practitioner role within the healthcare system. Assumes responsibility for own professional development by pursuing education, participating in professional committees and work groups, and contributing to a work environment where continual improvements in practice are pursued Employs consultative and leadership skills with intraprofessional and interprofessional teams to create change in health care and complex healthcare delivery systems. Participates in peer‐review activities e.g. publications, research, and practice Participates in relevant networks; regional, national, and international
Health Systems Leadership domain – Core competencies (*N* = 8) Contributes to development, implementation, and monitoring of organizational performance standards Assumes a leadership role of an inter professional healthcare team with a focus on the delivery of patient‐centred care and the evaluation of quality and cost‐effectiveness across the healthcare continuum Demonstrates a leadership role in enhancing group dynamics and managing group conflicts within the organization Plans and implements training and provides technical assistance and nursing consultation to health department staff, health providers, policy makers, and personnel in other community and governmental agencies and organizations Delegates and supervises tasks assigned to paraprofessional staff Creates a culture of ethical standards within organizations and communities Identifies internal and external issues that may impact delivery of essential medical and public health services Demonstrates working knowledge of the healthcare system and its component parts, including sites of care, delivery models, payment models, and the roles of healthcare professionals, patients, caregivers, and unlicensed professionals
Health Policy Leadership domain – Core competencies (*N* = 2) Guides, initiates, and provides leadership in policy‐related activities to influence practice, health services and public policy Articulates the value of nursing to key stakeholders and policy‐makers
Clinical and Health Systems Leadership domain – Core competencies (*N* = 4) Uses advanced communication skills/processes to lead quality improvement and patient safety initiatives in healthcare systems. Employs principles of business, finance, economics, and health policy to develop and implement effective plans for practice‐level and/or system‐wide practice initiatives that will improve the quality of care delivery. Advocates for and participates in creating an organizational environment that supports safe client care, collaborative practice and professional growth. Create positive healthy (work)environments and maintain a climate in which team members feel heard and safe
Professional and Health Systems Leadership domain – Core competencies (*N* = 1) Prepares through mentoring and coaching future generations of nurse leaders
Clinical, Health Systems and Health Policy Leadership domain – Core competencies (*N* = 1) Provides leadership in the evaluation and resolution of ethical and legal issues within healthcare systems relating to the use of information, information technology, communication networks, and patient care technology.

Seven studies and two frameworks reported on knowledge (Ailey et al., 2015; Bahouth et al., 2013; Carryer et al., 2007; The National Organization of Nurse Practotioner Faculties, 2014), skills (Ailey et al., 2015; Baernholdt & Cottingham, 2011; Maag et al., 2006; The National Organization of Clinical Nurse Specialists, 2010; The National Organization of Nurse Practotioner Faculties, 2014; Thompson & Nelson‐Marten, 2011) and attributes (Ailey et al., 2015; Bahouth et al., 2013). Both Ailey et al. (Ailey et al., 2015; Sievers & Wolf, 2006) and the NONPF (The National Organization of Nurse Practotioner Faculties, 2014) described skills and knowledge in terms of explicit curricula content for APNs. Other studies reported broadly formulated KSA. Eleven knowledge items, 21 skills and 21 attributes were identified (Table 3) and assigned to a leadership domain.

**Table 3:** Overview of identified needs for knowledge, skills, and attributes

	Leadership domain
Knowledge – the APN has knowledge of	
1. Legal and ethical dimensions of practice, policy directives and best practice guidelines that influence their own practice and the practice of the people they lead (Ailey et al., 2015; Bahouth et al., 2013; Carryer et al., 2007)	CL
2. Sciences/social sciences, disparities, social determinants (Ailey et al., 2015)	CL
3. Informatics (Ailey et al., 2015)	HS
4. Economics, policy, finance (Ailey et al., 2015)	HS, HP
5. Outcomes management and quality improvement (Ailey et al., 2015)	CL, HS
6. Collaboration with consumers and stakeholders (Ailey et al., 2015)	CL, HS
7. Inter professional leadership (The National Organization of Nurse Practotioner Faculties, 2014)	CL
8. Leadership positions in professional, political, or regulatory organizations (The National Organization of Nurse Practotioner Faculties, 2014)	HS, HP
9. Structure and functions of editorial/board roles (The National Organization of Nurse Practotioner Faculties, 2014)	All
10. Leadership, change, and management theories with application to practice (Ailey et al., 2015; The National Organization of Nurse Practotioner Faculties, 2014)	All
11. Political processes, political decision‐making processes, and healthcare advocacy (The National Organization of Nurse Practotioner Faculties, 2014)	HP
Skills – the APN shows skills to…	
1. Integrate care (Maag et al., 2006)	CL
2. Advocate for a client's interests (Maag et al., 2006)	CL
3. Apply evidence‐based practice, research/ standards of practice (Ailey et al., 2015)	CL
4. Critical thinking (Baernholdt & Cottingham, 2011)	All
5. Challenging current policies, procedures and practice environments using change theory and the theory of 6. Diffusion of dissemination. (Baernholdt & Cottingham, 2011)	HS, HP
6. Accessing, evaluating, and disseminating knowledge at the system level (Baernholdt & Cottingham, 2011)	HS
7. Reasoning to move from individual patient care concerns to group/population concerns and system solutions (Ailey et al., 2015)	HS
8. Systems thinking (The National Organization of Clinical Nurse Specialists, 2010)	All
9. Collaboration (The National Organization of Clinical Nurse Specialists, 2010)	All
10. Response to diversity (The National Organization of Clinical Nurse Specialists, 2010)	All
11. Clinical judgment (The National Organization of Clinical Nurse Specialists, 2010)	CL
12. Clinical enquiry (The National Organization of Clinical Nurse Specialists, 2010)	CL
13. Identify the need for change (Thompson & Nelson‐Marten, 2011)	CL
14. Design programs to facilitate behaviour change (Thompson & Nelson‐Marten, 2011)	CL
15. Persuade and encourage adoption of the change (Thompson & Nelson‐Marten, 2011)	All
16. Evaluate outcomes (Thompson & Nelson‐Marten, 2011)	CL
17. Synthesize the literature (Thompson & Nelson‐Marten, 2011)	PR
18. Problem solving	
a.Influencing and negotiation (Maag et al., 2006; The National Organization of Nurse Practotioner Faculties, 2014)	All
b.Conflict management (The National Organization of Nurse Practotioner Faculties, 2014)	All
c.Strategic thinking (The National Organization of Nurse Practotioner Faculties, 2014)	HS, HP
d.Managing change (The National Organization of Nurse Practotioner Faculties, 2014)	All
19. Communication	
a.Scholarly writing, manuscript, and abstract preparation (Baernholdt & Cottingham, 2011; Bahouth et al., 2013; The National Organization of Nurse Practotioner Faculties, 2014)	PR
b.Structuring and presenting persuasive arguments (Baernholdt & Cottingham, 2011; Bahouth et al., 2013; The National Organization of Nurse Practotioner Faculties, 2014)	All
20. Peer review	
a.Publications	
b.Presentations	
c.Research	
d.Practice (The National Organization of Nurse Practotioner Faculties, 2014)	PR
21. Leadership development	
Influence decision‐making bodies at the system, state, or national level (The National Organization of Nurse Practotioner Faculties, 2014)	HS, HP
Attributes – the APN*..*	
1. is champion of APN practice (Bahouth et al., 2013)	CL
2. is collaborative in issues that bridge nursing and medicine (Bahouth et al., 2013)	PR
3. is responsive to the needs of diverse stakeholders including the CEO, CFO, CMO, CNO, supervising physicians, and APNs. (Bahouth et al., 2013)	HS
4. is showing interaction modalities (Baernholdt & Cottingham, 2011)	All
5. has the ability to mentor APNs in professional development (Bahouth et al., 2013)	PR
6. is flexible in a transition from clinical role to executive policy decision‐making (Bahouth et al., 2013)	HP
7. is approachable by all levels of medical and nursing staff (Bahouth et al., 2013)	CL
8. is able to access key resources and relationships for the benefit of the APNs (Bahouth et al., 2013)	PR
9. is able to foster/translate research into practice and foster ongoing research (Bahouth et al., 2013)	CL
10. is articulate regarding advantages cost‐effective, quality care provided by APNs (Bahouth et al., 2013)	HS
11. is politically astute regarding organizational nuances, political and philosophical issues relative the APN role in relation to physician practice in the acute and critical care environment. (Bahouth et al., 2013)	HP
12. is known for previous experience in strategic planning, participation in executive policy, and decision‐making (Bahouth et al., 2013)	HS, HP
13. is known for quality leadership within the institution (Bahouth et al., 2013)	CL, HS
14. is aware of clinical leadership to leadership at micro and mezzo level (Ailey et al., 2015)	CL
15. is confident while advocating for the role of nursing (Sievers & Wolf, 2006)	PL, HS
16. is honest while advocating for the role of nursing (Sievers & Wolf, 2006)	PL, HS
17. is willing to take risk while advocating for the role of nursing (Sievers & Wolf, 2006)	PL, HS
18. solicited peer feedback (Sievers & Wolf, 2006)	CL
19. is open to learning new concepts (Sievers & Wolf, 2006)	CL
20. supports groups diversity and culture (Sievers & Wolf, 2006)	CL, HS
21. is able to articulate the CNS role and scope of practice to others (Sievers & Wolf, 2006)	HS

Note

Abbreviations: CL, clinical; PR, professional; HS, health systems; HP, health policy.

## 4 DISCUSSION

The results of this integrative review lead to the synthesis of 30 leadership competencies for APNs and CNLs derived from international literature and official documents of international nursing organizations. Competencies were furthermore designated to the clinical, professional, health systems or the health policy leadership domains, according to Hamric et al. (Hamric et al., 2014). Six competencies were linked to more than one domain. The clinical, professional and the health systems domains dominated regarding the number of competencies.

In the clinical leadership domain, core competencies are focused on delivering excellent patient care and concern items like collaboration with professionals and other health agencies, implementation of innovations, and enhancing EBP. Although EBP is often viewed as a stand‐alone competency (Hamric et al., 2014), leadership and EBP are strongly connected (Sastre‐Fullana et al., 2017). Stetler et al. (Stetler, Ritchie, Rycroft‐Malone, & Charns, 2014) assume supportive leadership as a key driver for the successful institutionalization of EBP in an organization (Stetler et al., 2014).

Competencies on the professional leadership domain appear to be clearly formulated and provide for sufficient direction to further develop the nursing profession. This is important because hospital decision makers need to learn from professionals about their roles and a collaborative evidence‐based vision on APN (Carter et al., 2013) (Kilpatrick et al., 2014; Kleinpell, 2013).

The leadership competencies in the Health Systems domain are shifting from direct patient care to the strategic level. Influencing at the strategic level requires an in‐depth understanding of healthcare systems to create and share an organizational vision on quality improvement, leading to the implementation of changes and to evaluate their results. (Thompson & Nelson‐Marten, 2011; Walker, Cooke, Henderson, & Creedy, 2011). Health system leadership also means that APNs and CNLs articulate the nursing perspective by joining or chairing interdisciplinary committees and raise their voice in the boardroom. However, formal positions for APNs and CNLs at strategic level are not self‐evident. System leadership can therefore only be reinforced when supported by managers and administrators of the organization (Hanson, 2015; Higgins et al., 2014).

Competencies related to the health policy domain were minimally present. Identified core competencies in the health policy domain were the guiding and initiating of leadership in policy‐related activities, to practice influence in health care and the articulation of the value of nursing to key stakeholders and policymakers on the (inter)national level. These rather abstract competencies do not allow for a clear understanding of the content and nature of health policy leadership. Further specification and operationalization are needed to guide nurses to the political arena. For example, health policy competences should be focussing on in‐depth understanding of global trends in relevant health issues and the profession's involvement in healthcare policy decisions (Rains & Barton‐Kriese, 2001). Additionally, information technology including e‐health applications and ‘Big Data’ analytics are important issues on the health policy agenda and the nursing perspective should be part of decision‐making processes in this area.

Half of the studies and two frameworks reported on KSA (table 3) needed for the development of leadership competencies. The distinction between KSA however, appeared somewhat unclear. Being knowledgeable about legal rules was described as an attribute in one study (Bahouth et al., 2013) and as knowledge in others (Ailey et al., 2015; Carryer et al., 2007). Although KSA are closely related to each other, a distinction is helpful to specify what is needed to achieve defined leadership competencies.

Acquiring leadership competencies and related KSA occurs over time and is comparable with Benner's continuum ‘from novice to expert’ (Benner, 1982). Both APNs and CNLs curricula and clinical learning programs should train and empower their students to become leaders. Evidenced‐based training programs for clinical, professional, and systems leadership are scarce (Elliott, Farnum, & Beauchesne, 2016b). Training programs for political leadership are even scarcer, which is in line with the identified competency gap in the health policy domain. The model laid out in this paper could provide a useful base for evidence‐based curriculum development, although identified competencies need to be further refined and discussed and completed with KSA related to each competency. Educational programs which integrate course work and clinical learning seem promising in developing and improving leadership competencies in especially the clinical and systems domains (Ailey et al., 2015; Sievers & Wolf, 2006; Thompson & Nelson‐Marten, 2011). Ainslie (Ainslie, 2017) advocates that organizations should map leadership competences to observable milestones so that progress can be clearly determined. This competence‐based learning has similarities with the concept of Entrustable Professional Activity (EPA). EPAs are elements of professional practice, that is, tasks or responsibilities that are observable and measurable in their process and outcome (Ten Cate, 2013) and may also be useful in developing leadership in APNs and CNLs. An assessment determines the entry competency levels and point out a personalized leadership development path. An APN, for example, may test at the expert level for ‘promoting and performing EBP’ but test at the novice level for ‘leading inter professional healthcare teams’. Additionally, situated coaching and mentoring is considered an essential element in educational and clinical learning programs (Ailey et al., 2015; Elliott, 2017).

Positive results are found for the effects of hierarchical leadership in nursing on quality of care and, more specifically, on nursing‐sensitive patient outcomes (Vaismoradi, Griffiths, Turunen, & Jordan, 2016; Wong, Cummings, & Ducharme, 2013). However, further research is needed to establish the relationship between leadership practices of APNs and CNLs and nursing‐sensitive patient outcomes (Dubois et al., 2017; Kapu & Kleinpell, 2013).

A limitation of this review is the fact that 24 of the 177 literature articles included based on title and abstract were not available in full text and the final selection of only 15 studies consisted of varying study designs and quality. Furthermore, most studies originated from the United States and Australia which might be challenging the representativeness of this review from an international perspective. Nonetheless, this review represents an integrative overview including a gap analysis of leadership competencies for APNs and CNLs in the current literature and as established by international nursing organizations.

## 5 CONCLUSION

This review identified 30 core leadership competencies for APNs and CNLs in the clinical, professional, health systems, and health policy leadership domains. The next steps include: (a) discussing gaps in this overview of competencies with master level‐educated nurses and educational institutes and linking KSA to each of the established leadership core competencies; (b) translating these competencies and aligned KSA to curricula and clinical learning programs; and (c) evaluating the effect of leadership competencies on nurse sensitive outcomes. These steps should be part of a continuous process needed for continuous quality improvement, healthcare reform, and high‐reliability health care.

## AUTHOR CONTRIBUTIONS

MH, CvO, JP, HV, AH: made substantial contributions to conception and design, or acquisition of data, or analysis and interpretation of data; MH, CvO, JP, HV, AH: Involved in drafting the manuscript or revising it critically for important intellectual content; MH, CvO, JP, HV, AH: Given final approval of the version to be published. Each author should have participated sufficiently in the work to take public responsibility for appropriate portions of the content; MH, CvO, JP, HV, AH: Agreed to be accountable for all aspects of the work in ensuring that questions related to the accuracy or integrity of any part of the work are appropriately investigated and resolved.

## References

[jan14092-bib-0001] American Association of Colleges of Nursing . (2004). Working Statement Comparing the Clinical Nurse Leadersm and Clinical Nurse Specialist Roles: Similarities, Differences and Complementarities. Washington, DC.

[jan14092-bib-0002] American Association of Colleges of Nursing . (2006). The Essentials of doctoral Education for Advanced Nursing Practice. Washington, DC.

[jan14092-bib-0003] American Association of Colleges of Nursing . (2007). White paper on the education and role of the clinical nurse leader. Retrieved from http://www.aacn.nche.edu/publications/white-papers/ClinicalNurseLeader.pdf

[jan14092-bib-0004] APRN Joint Dialogue Group . (2008). Consensus model for APRN regulation: licensure, accreditation, certification & education. APRN Joint Dialogue Group Report, July 7, 2008.

[jan14092-bib-0005] American Association of Colleges of Nursing . (2011). The Essentials of Master’s Education in Nursing. Washington, DC.

[jan14092-bib-0006] American Association of Colleges of Nursing . (2013). Master's essentials and clinical nurse leader competencies. Washington, DC.

[jan14092-bib-0007] AHPRA . (2014). Nursing and Midwifery Board of Australia. Nurse practitioner standards for practice, Nursing and Midwifery board of Australia. Melbourne VIC 3001. Retrieved from www.nursingmidwiferyboard.gov.au

[jan14092-bib-0008] Ailey, S. , Lamb, K. , Friese, T. , & Christopher, B. A. . (2015). Educating nursing students in clinical leadership. Nursing Management, 21(9), 23–28. 10.7748/nm.21.9.23.e1304 25629348

[jan14092-bib-0009] Ainslie, M. (2017). Dissertation. Competency based clinical education for advanced practice registered nurses: Raising the bar. Plymouth State University. New Hampshire.

[jan14092-bib-0010] Alimo‐Metcalfe, B. , & Alban‐Metcalfe, J. (2004). Leadership in public organisations. London, UK: Routledge Taylor & Francis group.

[jan14092-bib-0011] American Nurses Association (2013). Competency Model, Embark on the Journey. ANA Leadership Intitute.

[jan14092-bib-0012] Baernholdt, M. , & Cottingham, S. (2011). The Clinical Nurse Leader – new nursing role with global implications. International Nursing Review, 58(1), 74–78. 10.1111/j.1466-7657.2010.00835.x 21281297

[jan14092-bib-0013] Bahouth, M. N. , Ackerman, M. , Ellis, E. F. , Fuchs, J. , McComiskey, C. , Stewart, E. S. , & Thomson‐Smith, C. (2013). Centralized resources for nurse practitioners: Common early experiences among leaders of six large health systems. Journal of the American Association of Nurse Practitioners, 25(4), 203–212. 10.1111/j.1745-7599.2012.00793.x 24218238

[jan14092-bib-0014] Begley, C. , Murphy, K. , Higgins, A. , & Cooney, A. (2014). Policy‐makers' views on impact of specialist and advanced practitioner roles in Ireland: The SCAPE study. Journal of Nursing Management, 22(4), 410–422. 10.1111/jonm.12018 24809238

[jan14092-bib-0015] Bender, M. , Williams, M. , & Su, W. (2016). Diffusion of a nurse‐led healthcare innovation: Describing certified clinical nurse leader integration into care delivery. Journal of Nursing Administration, 46(7–8), 400–407. 27442903 10.1097/NNA.0000000000000365

[jan14092-bib-0016] Bender, M. , Williams, M. , Su, W. , & Hites, L. (2017). Refining and validating a conceptual model of Clinical Nurse Leader integrated care delivery. Journal of Advanced Nursing, 73(2), 448–464. 10.1111/jan.13113 27555500

[jan14092-bib-0017] Benner, P. (1982). From novice to expert. American Journal of Nursing, 82(3), 402–407. 6917683

[jan14092-bib-0018] Bolden, R. (2004). What is Leadership? University of Exeter, Centre for Leadership studies. Exeter.

[jan14092-bib-0019] Canadian Nurses Association . (2010). Canadian nurse practitioner core competency framework, Ottawa.

[jan14092-bib-0020] Carryer, J. , Gardner, G. , Dunn, S. , & Gardner, A. (2007). The core role of the nurse practitioner: Practice, professionalism and clinical leadership. Journal of Clinical Nursing, 16(10), 1818–1825. 10.1111/j.1365-2702.2007.01823.x 17880470

[jan14092-bib-0021] Carter, N. , Dobbins, M. , Ireland, S. , Hoxby, H. , Peachey, G. , & DiCenso, A. (2013). Knowledge gaps regarding APN roles: What hospital decision‐makers tell us. Nurs Leadersh (Tor Ont), 26(4), 60–75. 10.12927/cjnl.2013.23629 24377849

[jan14092-bib-0022] Delamaire, M. , & Lafortune, G. (2010). Nurses in advanced roles: A description and evaluation of experiences in 12 developed countries In OECD Health Working Papers (Vol. 54), Paris: OECD Publishing.

[jan14092-bib-0023] Dubois, C. A. , D'Amour, D. , Brault, I. , Dallaire, C. , Dery, J. , Duhoux, A. , … Zufferey, A. (2017). Which priority indicators to use to evaluate nursing care performance? A discussion paper. Journal of Advanced Nursing, 73(12), 3154–3167. 10.1111/jan.13373 28661049

[jan14092-bib-0024] Elliott, N. (2017). Building leadership capacity in advanced nurse practitioners ‐ the role of organisational management. Journal of Nursing Management, 25(1), 77–81. 10.1111/jonm.12444 27873383

[jan14092-bib-0025] Elliott, N. , Begley, C. , Sheaf, G. , & Higgins, A. (2016a). Barriers and enablers to advanced practitioners' ability to enact their leadership role: A scoping review. International Journal of Nursing Studies, 60, 24–45. 10.1016/j.ijnurstu.2016.03.001 27297366

[jan14092-bib-0026] Elliott, N. , Farnum, K. , & Beauchesne, M. (2016b). Utilizing team debate to increase student abilities for mentoring and critical appraisal of global health care in doctor of nursing practice programs. Journal of Professional Nursing, 32(3), 224–234. 10.1016/j.profnurs.2015.10.009 27216130

[jan14092-bib-0027] Falk‐Rafael, A. (2005). Speaking truth to power: Nursing's legacy and moral imperative. Advances in Nursing Science, 28(3), 212–223. 10.1097/00012272-200507000-00004 16106151

[jan14092-bib-0028] Fiedler, F. E. (1967). Leader style and accomplishment of groups acting together. Zeitschrift Fur Experimentelle Und Angewandte Psychologie, 14(2), 200–217. 5582359

[jan14092-bib-0029] Flemming, K. , Booth, A. , Hannes, K. , Cargo, M. , & Noyes, J. (2018). Cochrane Qualitative and Implementation Methods Group guidance series‐paper 6: Reporting guidelines for qualitative, implementation and process evaluation evidence syntheses. Journal of Clinical Epidemiology, 97, 79–85. 10.1016/j.jclinepi.2017.10.022 29222060

[jan14092-bib-0030] Gardner, G. , Carryer, J. , Gardner, A. , & Dunn, S. (2006). Nurse practitioner competency standards: Findings from collaborative Australian and New Zealand research. International Journal of Nursing Studies, 43(5), 601–610. 10.1016/j.ijnurstu.2005.09.002 16257407

[jan14092-bib-0031] Gerard, S. , Grossman, S. , & Godfrey, M. (2012). Course strategies for clinical nurse leader development. Journal of Professional Nursing, 28(3), 147–155. 10.1016/j.profnurs.2011.11.012 22640946

[jan14092-bib-0032] Goldberg, S. E. , Cooper, J. O. , Blundell, A. , Gordon, A. L. , Masud, T. , & Moorchilot, R. (2016). Development of a curriculum for advanced nurse practitioners working with older people with frailty in the acute hospital through a modified Delphi process. Age & Ageing, 45(1), 48–53. 10.1093/ageing/afv178 26764394

[jan14092-bib-0033] Gosling, J. , & Mintzberg, H. (2003). The five minds of a manager. Harvard Business Review, 81, 54–63. 14619151

[jan14092-bib-0034] Guillén, L. , & Saris, W. E. (2013). Competencies, personality traits, and organizational rewards of middle managers: A motive-based approach. Human Performance, 26(1), 66–92.

[jan14092-bib-0035] Hamric, A. , Hanson, C. , Tracy, M. , & O'Grady, E. (2014). Advanced Practice Nursing, An Integrative Approach. Philadelphia, PA: Elsevier Saunders.

[jan14092-bib-0036] Hamric, A. , Spross, J. , & Hanson, C. (2009). Advanced practice nursing : An integrative approach. Philadelphia, PA: Saundesr Elsevier.

[jan14092-bib-0037] Hanson, M. D. (2015). Role of the clinical nurse specialist in the journey to magnet recognition. AACN Advanced Critical Care, 26(1), 50–57 10.1097/NCI.0000000000000068 25594480

[jan14092-bib-0038] Higgins, A. , Begley, C. , Lalor, J. , Coyne, I. , Murphy, K. , & Elliott, N. (2014). Factors influencing advanced practitioners' ability to enact leadership: A case study within Irish healthcare. Journal of Nursing Management, 22(7), 894–905 12p 10.1111/jonm.12057 23879441

[jan14092-bib-0039] Hong, Q. N. , Gonzalez‐Reyes, A. , & Pluye, P. (2018). Improving the usefulness of a tool for appraising the quality of qualitative, quantitative and mixed methods studies, the Mixed Methods Appraisal Tool (MMAT). J Eval Clin Pract, 24(3), 459–467. 10.1111/jep.12884 29464873

[jan14092-bib-0040] Huber, M. , Knottnerus, A. , Green, L. , van der Hors, H. , Jadad, A. , Kromhout, D. , … Smid, H. (2011). How should we define health? BMJ, 343, d4163 10.1136/bmj.d4163 21791490

[jan14092-bib-0041] ICN . (2015). International Council of Nurses Leadership. For Change™ (LFC) program.

[jan14092-bib-0042] IOM (2011). The future of nursing: Leading change, Advancing Health. Washington, DC: Institute of Medicine.

[jan14092-bib-0043] Kalb, K. B. , Cherry, N. M. , Kauzloric, J. , Brender, A. , Green, K. , Miyagawa, L. , & Shinoda‐Mettler, A. (2006). A competency‐based approach to public health nursing performance appraisal. Public Health Nursing, 23(2), 115–138 24p 10.1111/j.1525-1446.2006.230204.x 16684187

[jan14092-bib-0044] Kapu, A. N. , & Kleinpell, R. (2013). Developing nurse practitioner associated metrics for outcomes assessment. Journal of the American Association of Nurse Practitioners, 25(6), 289–296. 10.1111/1745-7599.12001 24170592

[jan14092-bib-0045] Kilpatrick, K. , Kaasalainen, S. , Donald, F. , Reid, K. , Carter, N. , Bryant‐Lukosius, D. , … DiCenso, A. (2014). The effectiveness and cost‐effectiveness of clinical nurse specialists in outpatient roles: A systematic review. Journal of Evaluation in Clinical Practice, 20(6), 1106–1123. 10.1111/jep.12219 25040492

[jan14092-bib-0046] Kleinpell, R. (2013). Measuring outcomes in advanced practice nursing. Outcome assessment in advanced practice nursing. New York, NY: Springer Publishing Company.

[jan14092-bib-0047] Koolen, E. (2016). Competencies, attributes and traits: What’s the difference?. Retrieved from https://emilykoolen.com/2016/10/07/competencies-attributes-and-traits-whats-the-difference/

[jan14092-bib-0048] Kouzes, J. M. , & Posner, B. Z. (2012). The leadership challenge: How to Make Extraordinary Things Happen in Organizations. San: Francisco: Jossey‐Bass.

[jan14092-bib-0049] Leggat, S. G. , Balding, C. , & Schiftan, D. (2015). Developing clinical leaders: The impact of an action learning mentoring programme for advanced practice nurses. Journal of Clinical Nursing, 24(11‐12), 1576–1584. 10.1111/jocn.12757 25664819

[jan14092-bib-0050] Lynch, B. M. , McCormack, B. , & McCance, T. (2011). Development of a model of situational leadership in residential care for older people. Journal of Nursing Management, 19(8), 1058–1069. 10.1111/j.1365-2834.2011.01275.x 22074308

[jan14092-bib-0051] Maag, M. M. , Buccheri, R. , Capella, E. , & Jennings, D. L. (2006). A conceptual framework for a clinical nurse leader program. Journal of Professional Nursing, 22(6), 367–372. 10.1016/j.profnurs.2005.11.002 17141721

[jan14092-bib-0052] Moher, D. , Liberati, A. , Tetzlaff, J. , & Altman, D. G. & Group, P (2009). Preferred reporting items for systematic reviews and meta‐analyses: The PRISMA statement. PLoS Medicine, 6(7), e1000097. 19621072 10.1371/journal.pmed.1000097 PMC2707599

[jan14092-bib-0053] National Organization of Clinical Nurse Specialists. NACNS . (2010). Clinical Nurse Specialist Core Competencies. The National CNS competency taskforce.

[jan14092-bib-0054] Nelson‐Brantley, H. V. , & Ford, D. J. (2017). Leading change: A concept analysis. Journal of Advanced Nursing, 73(4), 834–846. 10.1111/jan.13223 27878849

[jan14092-bib-0055] Nieminen, A.‐L. , Mannevaara, B. , & Fagerström, L. (2011). Advanced practice nurses' scope of practice: A qualitative study of advanced clinical competencies. Scandinavian Journal of Caring Sciences, 25(4), 661–670 10p 10.1111/j.1471-6712.2011.00876.x 21371072

[jan14092-bib-0057] Northouse, P. G. (2014). Leadership: Theory and Practice (3rd, ed). Thousand Oaks, CA: Sage Publications.

[jan14092-bib-0058] O'Rourke, T. , & Higuchi, K. S. (2016). Activities and attributes of nurse practitioner leaders: Lessons from a primary care system change. Canadian Journal of Nursing Leadership, 29(3), 46–60. 10.12927/cjnl.2016.24892 28032835

[jan14092-bib-0059] Rains, J. W. , & Barton‐Kriese, P. (2001). Developing political competence: A comparative study across disciplines. Public Health Nursing, 18(4), 219–224. 10.1046/j.1525-1446.2001.00219.x 11468061

[jan14092-bib-0060] Sastre‐Fullana, P. , Morales‐Asencio, J. M. , Sesé‐Abad, A. , Bennasar‐Veny, M. , Fernandez‐Dominguez, J. , & DePedro‐Gomez, J. E. (2017). Advanced Practice Nursing Competency Assessment Instrument (APNCAI): Clinimetrci validation. British Medical Journal Open, 7, e013659. 10.1136/bmjopen-2016-013659 PMC5337725 28235968

[jan14092-bib-0061] Sievers, B. , & Wolf, S. (2006). Achieving clinical nurse specialist competencies and outcomes through interdisciplinary education. Clinical Nurse Specialist, 20(2), 75–80. 10.1097/00002800-200603000-00008 16609281

[jan14092-bib-0062] Stanley, J. M. , Gannon, J. , Gabuat, J. , Hartranft, S. , Adams, N. , Mayes, C. , … Burch, D. (2008). The clinical nurse leader: A catalyst for improving quality and patient safety. Journal of Nursing Management, 16(5), 614–622. 10.1111/j.1365-2834.2008.00899.x 18558932

[jan14092-bib-0063] Stetler, C. B. , Ritchie, J. A. , Rycroft‐Malone, J. , & Charns, M. P. (2014). Leadership for evidence‐based practice: Strategic and functional behaviors for institutionalizing EBP. Worldviews on Evidence‐Based Nursing, 11(4), 219–226. 10.1111/wvn.12044 24986669 PMC4240461

[jan14092-bib-0064] Ten Cate, O. (2013). Nuts and bolts of entrustable professional activities. Journal of Graduate Medical Education, 5(1), 157–158. 10.4300/JGME-D-12-00380.1 24404246 PMC3613304

[jan14092-bib-0056] The National Organization of Nurse Practotioner Faculties . (2014). Nurse Practitioner Core competencies. NONPF Report

[jan14092-bib-0065] Thompson, C. J. , & Nelson‐Marten, P. (2011). Clinical nurse specialist education: Actualizing the systems leadership competency. Clinical Nurse Specialist CNS, 25(3), 133–139. 10.1097/NUR.0b013e318217b5c5 21483244

[jan14092-bib-0066] Tong, A. , Flemming, K. , McInnes, E. , Oliver, S. , & Craig, J. (2012). Enhancing transparency in reporting the synthesis of qualitative research: ENTREQ. BMC Medical Research Methodology, 12, 181 10.1186/1471-2288-12-181 23185978 PMC3552766

[jan14092-bib-0067] Vaismoradi, M. , Griffiths, P. , Turunen, H. , & Jordan, S. (2016). Transformational leadership in nursing and medication safety education: A discussion paper. Journal of Nursing Management, 24(7), 970–980. 10.1111/jonm.12387 27144805

[jan14092-bib-0068] Vance, C. , & Larson, E. (2002). Leadership research in business and health care. Journal of Nursing Scholarship, 34(2), 165–171. 10.1111/j.1547-5069.2002.00165.x 12078542

[jan14092-bib-0069] Walker, R. , Cooke, M. , Henderson, A. , & Creedy, D. K. (2011). Characteristics of leadership that influence clinical learning: A narrative review. Nurse Education Today, 31(8), 743–756. 10.1016/j.nedt.2010.12.018 21255881

[jan14092-bib-0070] Whittemore, R. , & Knafl, K. (2005). The integrative review: Updated methodology. Journal of Advanced Nursing, 52(5), 546–553. 10.1111/j.1365-2648.2005.03621.x 16268861

[jan14092-bib-0071] WHO . (1948). Preamble to the constitution of the world health organization as adopted by the international health conference. New York, 19-22 June, 1946; signed on 22 July 1946 by the representatives of 61 States (Official Records of the World Health Organization, no. 2, p. 100) and entered into force on 7 April 1948.

[jan14092-bib-0072] Wong, C. A. , Cummings, G. G. , & Ducharme, L. (2013). The relationship between nursing leadership and patient outcomes: A systematic review update. Journal of Nursing Management, 21(5), 709–724. 10.1111/jonm.12116 23865924

