# Supplementary material for: An integrative review of leadership competencies and attributes in advanced nursing practice
Source: J Adv Nurs. 2019 Jul 21;75(11):2378–92. doi: 10.1111/jan.14092 (PMC6899698; doi:10.1111/jan.14092)
Supplement: Supplementary file 1 [file JAN-75-2378-s001.pdf]

Supplementary file 1 Search strategy

A1. Embase search strategy

Database: Embase
Search Strategy:
----
1 "carryer$".fc_auts. and "core role".fc_titl. and "2007".fc_pubyr. and "1818".fc_pg. (1)
2 "cummings$".fc_auts. and "factors contributing".fc_titl. and "2008".fc_pubyr. and "240".fc_pg. (1)
3 1 or 2 (2)
4 Leadership/ (41373)
5 leadership?.tw,kw. (26363)
6 exp Nurses/ (122391)
7 professional role/ or nurse's role/ (57768)
8 nurse?.tw,kw. (227412)
9 6 or 8 (274633)
10 4 or 5 (51625)
11 9 and 10 (8682)
12 professional competence/ or clinical competence/ (71247)
13 competenc*.tw,kw. (70085)
14 (leadership? adj5 factor?).tw,kw. (374)
15 "Attitude of Health Personnel"/ (64968)
16 attitude?.tw,kw. (125583)
17 or/12-16 (295066)
18 11 and 17 (2722)
19 "characteristics of nurse leaders".fc_titl. and "2012".fc_pubyr. (1)
20 *Leadership/ (15475)
21 leadership?.ti,kw. (9841)
22 20 or 21 (18384)
23 18 and 22 (1173)
24 ((clinical or professional or system?) adj3 leadership?).tw,kw. (1632)
25 23 and 24 (106)
26 25 (106)
27 limit 26 to yr="2000 -Current" (95)
28 (english or dutch).la. (22338513)
29 27 and 28 (95)
30 29 (95)
31 limit 30 to yr="2005 -Current" (77)
32 from 31 keep 1-70 (70)
33 *leadership/ (15475)
34 nursing/ (202671)
35 21 or 33 (18384)
36 8 or 34 (378486)
37 35 and 36 (3588)
38 13 or 14 or 16 (192581)
39 professional competence/ (25734)
40 health personnel attitude/ or nurse attitude/ (93635)
41 38 or 39 or 40 (289224)
42 37 and 41 (1194)
43 24 and 42 (113)

```
44    43 (113)
45    limit 44 to yr="2005 -Current" (89)
```

A2. Medline search strategy

```
Database: Ovid MEDLINE(R) In-Process & Other Non-Indexed Citations, Ovid
MEDLINE(R) Daily, Ovid MEDLINE(R) and Ovid OLDMEDLINE(R) <1946 to Present>
Search Strategy:
--------------------------------------------------------------------------------
1    "carryer$".fc_auts. and "core role".fc_titl. and "2007".fc_pubyr. and "1818".fc_pg. (1)
2    "cummings$".fc_auts. and "factors contributing".fc_titl. and "2008".fc_pubyr. and
"240".fc_pg. (1)
3    1 or 2 (2)
4    Leadership/ (28942)
5    leadership?.tw,kw. (23012)
6    exp Nurses/ (72052)
7    professional role/ or nurse's role/ (42979)
8    nurse?.tw,kw. (198076)
9    6 or 8 (235563)
10    4 or 5 (41245)
11    9 and 10 (7891)
12    professional competence/ or clinical competence/ (89081)
13    competenc*.tw,kw. (55971)
14    (leadership? adj5 factor?).tw,kw. (327)
15    "Attitude of Health Personnel"/ (95418)
16    attitude?.tw,kw. (105489)
17    or/12-16 (298295)
18    11 and 17 (2707)
19    "characteristics of nurse leaders".fc_titl. and "2012".fc_pubyr. (1)
20    *Leadership/ (15384)
21    leadership?.ti,kw. (8770)
22    20 or 21 (17719)
23    18 and 22 (1128)
24    ((clinical or professional or system?) adj3 leadership?).tw,kw. (1301)
25    23 and 24 (101)
26    25 (101)
27    limit 26 to yr="2000 -Current" (90)
28    (english or dutch).la. (20372595)
29    27 and 28 (90)
30    29 (90)
31    limit 30 to yr="2005 -Current" (70)
```

CINAHL

#	Query
S12	S7 AND S11
S11	S8 OR S9 OR S10
S10	TI ((leader* N5 (characteristic* or competenc* or attitude* or attribute* or factor*))) OR AB ((leader* N5 (characteristic* or competenc* or attitude* or attribute* or factor*)))
S9	TI competenc* OR AB competenc*
S8	(MH "Professional Competence+") OR (MH "Clinical Competence+") OR (MH "Competency Assessment")
S7	S3 AND S6
S6	S4 OR S5
S5	TI nurs* OR AB nurs*
S4	(MH "Nurses+")
S3	S1 OR S2
S2	TI leadership* OR AB leadership*
S1	(MH "Leadership")

Supplementary file 2 Quality Appraisal of included studies (15)

**Quality Appraisal of Mixed methods studies**							
First Author, Publication year	S1. Are there clear research questions?	S2. Do the collected data allow to address the research questions?	1.Is there an adequate rationale for using a mixed methods design to address the research question?	2.Are the different components of the study effectively integrated to answer the research question?	3.Are the outputs of the integration of qualitative and quantitative components adequately interpreted?	4.Are divergences and inconsistencies between quantitative and qualitative results adequately addressed?	5.Do the different components of the study adhere to the quality criteria of each tradition of the methods involved?
Bahouth 2011	Yes	Yes	Yes	No	No	No	No
Comments: (Journal of the American Association of Nurse Practitioner) Outcomes of the survey and focus groups are described adequately including leadership competencies for hospital-based NP practice. However, there is no clear description of the data collection and data analysis process.							
Bender 2016	Yes	Yes	Yes	Yes	Yes	Yes	Yes
Comments: (Journal of Advanced Nursing) The research question could only be answered by using a mixed methods design. The authors effectively integrated the different component of the study by 1) using focus groups and a Delphi study for refining a preliminary CNL practice model and to develop a survey to test this model, and 2) subsequently test the model quantitatively by using the survey. The quantitative analysis lead to an adaptation of the practice model. The components of the study adhere to the quality criteria of each tradition in terms of sampling, data collection and data analysis.							
Goldberg 2016	Yes	Yes	Yes	Yes	Yes	Yes	Yes
Comments: (Age and Ageing) Based on a literature review, 69 APN competencies were judged by 31 experts in three Delphi rounds. The research question was clear, and the collected data and sampling strategy were adequate to answer the research question. The authors described the methodology systematically: 1) the level of agreement was analyzed by a predefined process and 2) a flowchart of agreement of competencies was given. Non-response was low.							
**Quality Appraisal of Qualitative studies**							
First Author, Publication year	S1. Are there clear research questions?	S2. Do the collected data allow to address the research questions?	1.Is the qualitative approach appropriate to answer the research question?	2.Are the qualitative data collection methods adequate to address the research question?	3.Are the findings adequately derived from the data?	4. Is the interpretation of results sufficiently substantiated by data?	5. Is there coherence between qualitative data sources, collection, analysis and interpretation?
Carryer 2007	Yes	Yes	Yes	Yes	Yes	Yes	Yes
Comments: (Journal of Clinical Nursing) This paper refers to a study also described by Gardner et al. 2006. The primary source of data for this component of the study was interviews with participating NPs. The authors have identified three major conceptual categories that are identified as the practice domains that describe the core role of the NP. However, the results of the additional data sources, mentioned in the introduction, are not presented in this paper.							
Nieminen 2011	Yes	Yes	Yes	Yes	Yes	Yes	Yes
Comments: (Caring Sciences) Seven focus group interviews of Clinical Nurse specialists and APN students. The research question for this sub study was clear and the approach, collected data, analysis and interpretation were appropriate and adequate.							
O'Rourke 2016	Yes	Yes	Yes	Yes	Yes	Yes	Yes
Comments: (Nursing Leadership) This study was part of a larger case study that examined stakeholder participation in a system change led by NPs. The change involved the introduction of a new NP-led model of team-based primary care delivery in Ontario. The research question for this sub study was clear and the approach, collected data, analysis and interpretation were appropriate and adequate.							
**Quality Appraisal of Non-Randomized studies**							
First Author, Publication year	S1. Are there clear research questions?	S2. Do the collected data allow to address the research questions?	1. Are the participants representative of the target population?	2. Are measurements appropriate regarding both the outcome and intervention (or exposure)?	3. Are there complete outcome data?	4. Are the confounders accounted for in the design and analysis?	5. During the study period, is the intervention administered (or exposure occurred) as intended?

Leggat 2015	Yes	Yes	Yes	Yes	Yes	Can't tell	Yes
Comments: (Journal of Clinical Nursing) This study's focus is Clinical leadership, with a duration of 18 months. Coaching and action learning were used. A questionnaire was used for measures of self reported leadership practices (LPI K&P) + one closed and two (qualitative) open ended questions. The research question was clear, and the collected data and sampling strategy were adequate to answer the research question. The intervention is described. Reporting of the quantitative data is adequate. The study has a small sample size and only uses the self reported questionnaire. Also, there is no clear description of the data analysis process of the open ended questions added to this questionnaire.							
**Quality Appraisal N/A**							
First Author, Publication year	S1. Are there clear research questions?	S2. Do the collected data allow to address the research questions?					
Ailey 2015	No	-	-	-	-	-	-
Comments: (Nursing Management) Utilizing the MSLNL the authors developed a clinical leadership program aimed at improving quality care and clinical care systems for patients with intellectual and developmental disabilities (IDDs).The theoretical framework is described including Leadership competencies, attributes and curriculum elements.The course and output – ten student final leadership projects involving 22 students are described. Experiences are not elicited and described in according to case study formats.							
Bearnholdt 2011	No	-	-	-	-	-	-
Comments: (International Nursing Review) The authors analyzed three key reports to describe the development of the CNL's role and education, the CNL's impact and potential to improve quality globally. The authors listed Health Professions Core Competencies and described the Essential CNL Curriculum Components.							
Gardner 2006	No	-	-	-	-	-	-
Comments: (International Journal of Nursing Studies) Although a comprehensive overview of NP competencies and performance indicators is given, the report does not describe the research methodology used and related analytical and interpretive processes. Concerning insight in research methodology, the reader is directed to the ANMC report (Gardner et al., 2004) for details of research results.							
Gerard 2012	No	-	-	-	-	-	-
Comments: (Journal of Professional Nursing) Utilizing the AACN CNL white paper and published resources faculty developed a clinical leadership course focused on active learning and reflection. The first year is evaluated based on experience of nine students. Experiences are not elicited and described in a systematic way according to qualitative or quantitative research standards.							
Maag 2006	No	-	-	-	-	-	-
Comments: (Journal of professional Nursing) Transition Theory and Symptom management model are used but there is no clear description of how the model was developed; following a literature review and analysis of the American Association of Colleges of Nursing (AACN) description of the CNL. Choices are not supported by explicit rationales.							
Kalb 2006	No	-	-	-	-	-	-
Comments: (Public health Nursing) Performance elements were described for all eight competencies and for each of five nursing classifications.<br>Development is described, pilot testing among 50 nurses is not described, a small pilot test among supervisors was used in the development phase. Participants at different levels were positive about the tool, and about the included goal setting part. This pilot test was not thoroughly conducted among the 50 nurses, in short an indication of first results are presented.							
Thompson 2011	No	-	-	-	-	-	-
Comments: (Public Health Nursing) An evidence-based change project - as the capstone project in the CNS master's program – was described, facilitating in developing systems leadership and change agent skills following the CNS core competencies developed by the National Association of Clinical Nurse Specialists (NACNS). A detailed description of the project phases and corresponding activities is given.							
Sievers 2006	No	-	-	-	-	-	-
Comments: (Clinical Nurse Specialist) The authors described a quality improvement initiative to create an interdisciplinary educational experience for clinical nurse specialist (CNS) students and postgraduate physicians, following the CNS core competencies developed by the National Association of Clinical Nurse Specialists (NACNS). Findings were described following the core competency framework and richly illustrated.							

Supplementary file 3 Identified competencies from the literature linked to the core competencies (CC) in table 2

Core Competency nr. (table 2)	Core competencies identified from the literature
1	1.1 Provides leadership in the management of clinical care and is a resource person, educator and role model (CNA, 2010)<br>1.2 Provide leadership to the healthcare team to promote health, facilitate self-care management, optimize patient engagement and prevent future decline including progression to higher levels of care and readmissions (AACN, 2013)<br>1.3 Acts as a preceptor, mentor and coach to nursing colleagues, other members of the health-care team and students (CNA, 2010)<br>1.4 Demonstrates leadership that uses critical and reflective thinking (NONPF, 2014)<br>1.5 Risk anticipation for individuals and cohorts of patients (AACN, 2013)<br>1.6 Team leadership, management and collaboration with other health professional team members (AACN, 2013)<br>1.7 Advocacy for patients, communities, and the health professional team (AACN, 2013)<br>1.8 Demonstrates the ability to engender trust so that staff feel confident about sharing difficult problems and feel able to point out deficiencies in care at an early stage (Goldberg et al., 2016)<br>1.9 Demonstrates the ability to identify with senior team members and support other members of the team to do the same: situations where consideration of medical legal matters may be of benefit (Goldberg et al., 2016)<br>1.10 Demonstrates the ability to identify with senior team members and support other members of the team to do the same: Cases which should be reported to external bodies and where appropriate initiate that report (Goldberg et al., 2016)<br>1.11 Coordinates the care of patients with use of system and community resources to assure successful health/illness/wellness transitions, enhance delivery of care, and achieve optimal patient outcomes (NACNS, 2010)
2.	2.1 Assumes as a clinical expert, a leadership role in establishing and monitoring standards of practice to improve client care (Kalb et al., 2006)<br>2.2 Participates in quality management activities using quality improvement and evaluation approaches (Kalb et al., 2006)<br>2.3 Monitors own practice as well as participates in intra- and inter-disciplinary peer supervision and review (Gardner, Carryer, Gardner, & Dunn, 2006)<br>2.4 Evaluates the outcomes of own practice (AHPRA, 2014)<br>2.5 Demonstrates the skills to evaluate own performance and the service in which they work (Goldberg et al., 2016)<br>2.6 Demonstrates contribution to service development (Goldberg et al., 2016)<br>2.7 Demonstrates the development of leadership skills to improve services for older people (Goldberg et al., 2016)<br>2.8 Demonstrates the ability to act as a resource in the design and development of older adult services in hospital and the community (Goldberg et al., 2016)<br>2.9 Demonstrates the ability to provide support to junior members of the multidisciplinary team including doctors, nurses and therapists (Goldberg et al., 2016)<br>2.10 Demonstrates that they are professional, compassionate and approachable, have high standards of care, good organizational, interpersonal and communication skills and the ability to cope with the demands placed on them (Goldberg et al., 2016)<br>2.11 Develops age-specific clinical standards, policies and procedures (NACNS, 2010)
3.	3.1 Analyzes organizational systems for barriers and promotes enhancements that affect client health care status. (Kalb et al., 2006)
4.	4.1 Identifies current relevant scientific health information and applies it appropriately to public health practice (Kalb et al., 2006)<br>4.2 Participates in research and demonstration projects that seek to improve the health of individuals and populations, and determine new ways to address health issues (Kalb et al., 2006)<br>4.3 DNP graduates engage in advanced nursing practice and provide leadership for evidence-based practice. This requires competence in knowledge application activities: the translation of research in practice, the evaluation of practice, improvement of the reliability of health care practice and outcomes, and participation in collaborative research. (AACN, 2006)<br>4.4 Evaluate the efficacy and utility of evidence-based care delivery approaches and their outcomes at the micro system level.(AACN, 2013)<br>4.5 Develop and evaluate care delivery approaches that meet current and future needs of patient populations based on scientific findings in nursing and other clinical sciences, as well as organizational, political, and economic sciences.(AACN, 2006)<br>4.6 DNP graduates engage in leadership to integrate and institutionalize evidence-based clinical prevention and population health services for individuals, aggregates, and populations. Current concepts of public health, health promotion, evidence-based recommendations, determinants of health, environmental/occupational health, and cultural diversity and sensitivity guide the practice of DNP graduates. (AACN, 2006)<br>4.7 Lateral integration of care for individuals and cohorts of patients (AACN, 2013)<br>4.8 Design and implementation of evidence-based practice(s) (AACN, 2013)<br>4.9 Demonstrates engagement in audit and participation in the design and implementation of evidence-based protocols and processes of care to reduce adverse events common to older adults

	(Goldberg et al., 2016)<br>4.10 Uses effective strategies for changing clinician and team behavior to encourage adoption of evidence-based practices and innovations in care delivery (NACNS, 2010)<br>4.11 Leads system change to improve health outcomes through evidence based practice: (c) Facilitates the adoption of practice change (NACNS, 2010)<br>4.12 Provides leadership for collaborative, evidence-based revision of diagnoses and plans of care, to improve patient outcomes (NACNS, 2010)
5.	5.1 Provides leadership and acts as a liaison with other community agencies and professionals, advocates on behalf of vulnerable individuals and populations, participates in assessing and evaluating health care services to ensure that people are informed of available programs and services and are assisted in the utilization of those services (Kalb et al., 2006)<br>5.2 Demonstrates understanding that root causes analysis exists as a strategy for learning from mistakes (Goldberg et al., 2016)<br>5.3 The CNL facilitates communication between nurses, patients and other professions at the point of care (Bender, Williams, Su, & Hites, 2017)<br>5.4 The CNL is a communication 'hub' between multi-professional clinicians and patients at the point of care (Bender et al., 2017)<br>5.5 The CNL facilitates/develops effective and meaningful nursing and inter-professional rounds (Bender et al., 2017)<br>5.6 The CNL creates interactive relationships between professions (Bender et al., 2017)
6.	6.1 Engages in and leads clinical collaborations that optimise outcomes for patients/clients/communities (Gardner et al., 2006)<br>6.2 Actively participates as a senior member and/or leader of relevant multi-disciplinary teams (Gardner et al., 2006)<br>6.3 Collaborate with healthcare professionals, including physicians, advanced practice nurses, nurse managers and others, to plan, implement and evaluate an improvement opportunity. (AACN, 2013)<br>6.4 Assume a leadership role, in collaboration with other inter professional team members, to facilitate transitions across care settings to support patients and families and reduce avoidable recidivism to improve care outcomes.(AACN, 2013)<br>6.5 The CNL facilitates multi-professional communication to gather clinical information (Bender et al., 2017)<br>6.6 The CNL reaches out to all multi-professional clinicians at the point of care (Bender et al., 2017)<br>6.7 The CNL builds relationships with everyone who supports the patient (Bender et al., 2017)<br>6.8 The CNL builds relationships with all multi-professional clinicians involved with patient care (Bender et al., 2017)<br>6.9 Provides leadership in promoting interdisciplinary collaboration to implement outcome-focused patient care programs meeting the clinical needs of patients, families, populations and communities (NACNS, 2010)<br>6.10 Provides leadership for establishing, improving, and sustaining collaborative relationships to meet clinical needs (NACNS, 2010)
7.	7.1 Demonstrates support of the public health mission to protect and promote the health of all residents through implementing primary prevention strategies that prevent health problems from starting, spreading, or progressing (Kalb et al., 2006)<br>7.2 Performs in a manner consistent with site/ organizational productivity goals (Kalb et al., 2006)<br>7.3 Aligns practice with overall organizational goals (Kalb et al., 2006)<br>7.4 Helps create key values and shared vision and uses these principles to guide action (Kalb et al., 2006)
8.	8.1 Advances practice through the development and implementation of innovations incorporating principles of change (NONPF, 2014)<br>8.2 Assumes complex and advanced leadership roles to initiate and guide change (NONPF, 2014)<br>8.3 Guides, initiates and provides leadership in the development and implementation of standards, practice guidelines, quality assurance, and education and research initiatives.(CNA, 2010)<br>8.4 Clinical leadership for patient-care practices and delivery, including the design, coordination, and evaluation of care for individuals, families, groups, and populations. (AACN, 2013)<br>8.5 Provides leadership in planning data collection and quality monitoring (NACNS, 2010)
9.	9.1 Participates in professional organizations and activities that influence advanced practice nursing (NONPF, 2014)<br>9.2 Influencing curricula changes. (ICN, 2015)
10.	10.1 Articulates and promotes the nurse practitioner role in clinical, political and professional contexts. (Gardner et al., 2006)<br>10.2 Communicates practice knowledge effectively both orally and in writing. (NONPF, 2014)<br>10.3 Provides leadership in the development and integration of the nurse practitioner role within the health-care system.(CNA, 2010)<br>10.4 Articulates and promotes the role of the nurse practitioner to clients, other health-care providers, social and public service sectors, the public, legislators and policy-makers.(CNA, 2010)<br>10.5 Align the contribution of the profession in socioeconomic, professional and regulatory developments. (ICN, 2015)
11.	11.1 Assumes responsibility for own professional development by pursuing education, participating in professional committees and work groups, and contributing to a work environment where continual improvements in practice are pursued. (Kalb et al., 2006)<br>11.2 Seeks professional certification in the area of expertise when available (Kalb et al., 2006)<br>11.3 Maintain and advance their own competence and to identify, develop and support future generations of leaders. (ICN, 2015)

12.	12.1 Employ consultative and leadership skills with intraprofessional teams to create change in health care and complex healthcare delivery systems. (AACN, 2006)
13.	13.1 Participates in peer-review activities as appropriate. (Kalb et al., 2006)
14.	14.1 Networking nationally, regionally and internationally. (ICN, 2015)<br>14.2 Participate in relevant networks. (ICN, 2015)
15.	15.1 Contributes to development, implementation, and monitoring of organizational performance standards (Kalb et al., 2006)<br>15.2 Use systems theory in the assessment, design, delivery, and evaluation of health care within complex organizations. (AACN, 2013)<br>15.3 Sustaining development. (ICN, 2015)<br>15.4 Develop, implement and evaluate projects (ICN, 2015)<br>15.5 Participation in identification and collection of care outcomes (AACN, 2013)<br>15.6 Accountability for evaluation and improvement of point-of-care outcomes, including the synthesis of data and other evidence to evaluate and achieve optimal outcomes (AACN, 2013)<br>15. 7 Information management or the use of information systems and technologies to improve healthcare outcomes (AACN, 2013)<br>15.8 Determines nursing practice and system interventions that will promote patient, family and community safety (NACNS, 2010)<br>15.9 Provides leadership in the design, implementation and evaluation of process improvement initiatives (NACNS, 2010)
16.	16.1 Uses the legal and political systems to effect change (Kalb et al., 2006)<br>16.2 Helps create key values and shared vision and uses these principles to guide action (Kalb et al., 2006)<br>16.3 Assume a leadership role of an interprofessional healthcare team with a focus on the delivery of patient-centered care and the evaluation of quality and cost-effectiveness across the healthcare continuum (AACN, 2013)<br>16.4 Leadership and management in health services (ICN, 2015)<br>16.5 Developing quality cost effective health services (ICN, 2015)<br>16.6 Contributing within the broader health and management teams (ICN, 2015)<br>16.7 The CNL identifies all participants involved in a point of care process (Bender et al., 2017)<br>16.8 The CNL builds teams to improve care processes (Bender et al., 2017)<br>16.7 The CNL brings people together from all the disciplines and departments affected by a care process to work together to improve the process (Bender et al., 2017)<br>16.8 The CNL is a resource for teamwork (Bender et al., 2017)<br>16.9 The CNL is a consistently present role model for all staff working at the point of care (Bender et al., 2017)<br>16.10 The CNL helps staff identify and create solutions for patient care needs (Bender et al., 2017)<br>16.11 Facilitates the provision of clinically competent care by staff/team through education, role modeling, teambuilding, and quality monitoring (NACNS, 2010)<br>16.12 Considers fiscal and budgetary implications in decision making regarding practice and system modifications:<br>(a) Evaluates use of products and services for appropriateness and cost/benefit in meeting care needs (NACNS, 2010)<br>(b) Conducts cost/benefit analysis of new clinical technologies (NACNS, 2010)<br>(c) Evaluates impact of introduction or withdrawal of products, services, and technologies (NACNS, 2010)<br>16.13 Leads system change to improve health outcomes through evidence based practice:<br>(a) Specifies expected clinical and system level outcomes (NACNS, 2010)<br>(b) Designs programs to improve clinical and system level processes and outcomes (NACNS, 2010)<br>16.14 Evaluates impact of CNS and other nursing practice on systems of care using nurse-sensitive outcomes (NACNS, 2010)<br>16.15 Disseminates outcomes of system-level change internally and externally (NACNS, 2010)<br>16.16 Provides leadership in the system-wide implementation of quality improvements and innovations (NACNS, 2010)
17.	17.1 Leading organizational and environmental stability (leadership in a learning and caring culture). (Nieminen, Mannevaara, & Fagerström, 2011)<br>17.2 Be effective and dynamic leaders and managers (ICN, 2015)<br>17.3 Demonstrate a leadership role in enhancing group dynamics and managing group conflicts. (AACN, 2013)<br>17.4 Demonstrates the ability to manage conflict appropriately, following the local hospital protocols (Goldberg et al., 2016)<br>17.5 Uses leadership, team building, negotiation, and conflict resolution skills to build partnerships within and across systems, including communities (NACNS, 2010)<br>17.6 Provides leadership in conflict management and negotiation to address problems in the healthcare system (NACNS, 2010)

18.	18.1 Plans and implements training and provides technical assistance and nursing consultation to health department staff, health providers, policy makers, and personnel in other community and governmental agencies and organizations (Kalb et al., 2006)
19.	19.1 Delegates and supervises tasks assigned to paraprofessional staff (Kalb et al., 2006)<br>19.2 The CNL provides ongoing support for staff to lead their own practice (Bender et al., 2017)
20.	20.1 Creates a culture of ethical standards within organizations and communities (Kalb et al., 2006)<br>20.2 Demonstrates the ability to advocate to create/enhance positive, health promoting environments and maintain a climate of dignity, compassion and privacy (Goldberg et al., 2016)
21.	21.1 Identifies internal and external issues that may impact delivery of essential medical and public health services (Kalb et al., 2006)<br>21.2 Performs system level assessments to identify variables that influence nursing practice and outcomes, including but not limited to:<br>(a) Population variables (age distribution, health status, income distribution, culture) (NACNS, 2010)<br>(b) Environment (schools, community support services, housing availability, employment opportunities) (NACNS, 2010)<br>(c) Internal and external political influences/stability (NACNS, 2010)
22.	22.1 Evaluates the impact of social factors (such as literacy, poverty, domestic violence and racial attitudes) on the health of individuals and communities and acts to moderate the influence of these factors on the specific population/individual (Gardner et al., 2006)<br>22.2 Demonstrate business and economic principles and practices, including cost-benefit analysis, budgeting, strategic planning, human and other resource management, marketing, and value-based purchasing. (AACN, 2013)<br>22.3 Demonstrate working knowledge of the healthcare system and its component parts, including sites of care, delivery models, payment models, and the roles of health care professionals, patients, caregivers, and unlicensed professionals. (AACN, 2013)<br>22.4 Performs system level assessments to identify variables that influence nursing practice and outcomes, including but not limited to:<br>(a) System of health care delivery (NACNS, 2010)<br>(b) Regulatory requirements (NACNS, 2010)<br>(c) Health care financing (NACNS, 2010)<br>(d) Recurring practices that enhance or compromise patient or system outcomes (NACNS, 2010)
23.	*23.1 Engages in and leads informed critique and influence at the systems level of health care*<br>(a) Critiques the implication of emerging health policy on the nurse practitioner role and the client population (Gardner et al., 2006)<br>*23.2 Engages in and leads informed critique and influence at the systems level of health care*<br>(c) Maintains current knowledge of financing of the health-care system as it affects delivery of care (Gardner et al., 2006)<br>*23.3 Engages in and leads informed critique and influence at the systems level of health care*<br>(d) Influences health-care policy and practice through leadership and active participation in workplace and professional organizations and at state and national government levels (Gardner et al., 2006)<br>*23.4 Engages in and leads informed critique and influence at the systems level of health care*<br>(e) Actively contributes to and advocates for the development of specialist, local and national health-service policy that enhances nurse practitioner practice and health of the community (Gardner et al., 2006)<br>23.5 Makes presentations to care key policy makers, health care professionals, specific populations, funding sources, and more general audiences in order to raise awareness and/or secure collaboration and funding (Kalb et al., 2006)<br>23.6 Guides, initiates and provides leadership in policy-related activities to influence practice, health services and public policy (NONPF, 2014)<br>23.7 Demonstrate leadership in the development and implementation of institutional, local, state, federal, and/or international health policy. (AACN, 2006)<br>23.8 Health planning, health and social policy development, and political skills. (ICN, 2015)<br>23.9 Engage in the development of health and social policy (ICN, 2015)<br>23.10 Contribute effectively to public sector and health reform in their country. (ICN, 2015)
24.	24.1 Contribute to budget development at the micro system level. (AACN, 2013)<br>24.2 Articulate the value of nursing and other health professionals to key stakeholders and policy-makers. (ICN, 2015)<br>24.3 Makes presentations to key policy makers, health care professionals, specific populations, funding sources, and more general audiences in order to raise awareness and/or secure collaboration and funding (Bender et al., 2017)

25.	25.1 Establishes effective communication strategies that promote positive multi-disciplinary clinical partnerships (Gardner et al., 2006)<br>25.2 Ensure accountability for quality of health care and patient safety for populations with whom they work.<br>(a) Use advanced communication skills/processes to lead quality improvement and patient safety initiatives in health care systems. (AACN, 2006)<br>25.3 The CNL creates communication tools, such as electronic databases or care guidelines (Bender et al., 2017)<br>25.4 The CNL facilitates effective ways to communicate information to all point of care multi-professional clinicians (Bender et al., 2017)
26.	26.1 Participates in efforts care to minimize costs and unnecessary duplication of testing or other diagnostic activities and to facilitate timely treatment of the patient (Kalb et al., 2006)<br>26.2 Ensure accountability for quality of health care and patient safety for populations with whom they work.<br>(b) Employ principles of business, finance, economics, and health policy to develop and implement effective plans for practice-level and/or system-wide practice initiatives that will improve the quality of care delivery. (AACN, 2006)<br>26.3 Ensure accountability for quality of health care and patient safety for populations with whom they work.<br>(c) Develop and/or monitor budgets for practice initiatives. (AACN, 2006)<br>26.4 Ensure accountability for quality of health care and patient safety for populations with whom they work.<br>(d) Analyze the cost-effectiveness of practice initiatives accounting for risk and improvement of health care outcomes.(AACN, 2006)
27	27.1 The CNL is a responsive/available resource to staff based on their needs at the moment (Bender et al., 2017)<br>27.2 The CNL empowers staff nurses to perform to their full scope of practice (Bender et al., 2017)<br>27.3 Provides leadership in maintaining a supportive and healthy work environment (NACNS, 2010)
28.	28.1 Preparing through mentoring and coaching future generations of nurse and non-nurse leaders. (ICN, 2015)<br>28.2 Prepare other nurses and health professionals for top management roles in nursing and health services. (ICN, 2015)
29.	29.1 Advocates for, participates in, or leads systems that support safe care, partnership and professional growth. (AHPRA, 2014)<br>29.2 Advocates for improved access, quality and cost effective health care. (NONPF, 2014)<br>29.3 Provides leadership to foster collaboration with multiple stakeholders (e.g. patients, community, integrated health care teams, and policy makers) to improve health care. (NONPF, 2014)<br>29.4 Advocates for and participates in creating an organizational environment that supports safe client care, collaborative practice and professional growth. (CNA, 2010)<br>29.5 Stewardship and leveraging of human, environmental, and material resources (AACN, 2013)
30.	30.1 Provide leadership in the evaluation and resolution of ethical and legal issues within healthcare systems relating to the use of information, information technology, communication networks, and patient care technology. (AACN, 2006)<br>30.2 Ensure accountability for quality of health care and patient safety for populations with whom they work.<br>(e) Demonstrate sensitivity to diverse organizational cultures and populations, including patients and providers. (AACN, 2006)<br>30.3 Develop and/or evaluate effective strategies for managing the ethical dilemmas inherent in patient care, the health care organization, and research. (AACN, 2006)

AACN. (2006). *The Essentials of doctoral Education for Advanced Nursing Practice*. Retrieved from

AACN. (2013). *Master's Essentials and Clinical Nurse Leader Competencies*. Retrieved from www.aacn.nche.edu/cnl/CNL-competencies-October-2013.pdf

AHPRA. (2014). Nurse practitioner standards for practice, Nursing and Midwifery board of Australia.

Bender, M., Williams, M., Su, W., & Hites, L. (2017). Refining and validating a conceptual model of Clinical Nurse Leader integrated care delivery. *Journal of Advanced Nursing*, *73*(2), 448-464. doi:https://dx.doi.org/10.1111/jan.13113

CNA, C. N. A. (2010). Canadian Nurse Practitioner Core Competency Framework.

Gardner, G., Carryer, J., Gardner, A., & Dunn, S. (2006). Nurse practitioner competency standards: findings from collaborative Australian and New Zealand research. *International Journal of Nursing Studies*, *43*(5), 601-610 610p.

Goldberg, S. E., Cooper, J. O., Blundell, A., Gordon, A. L., Masud, T., & Moorchilot, R. (2016). Development of a curriculum for advanced nurse practitioners working with older people with frailty in the acute hospital through a modified Delphi process. *Age & Ageing, 45*(1), 48-53. doi:10.1093/ageing/afv178

ICN. (2015). International Council of Nurses Leadership For Change™ (LFC) program.

Kalb, K. B., Cherry, N. M., Kauzloric, J., Brender, A., Green, K., Miyagawa, L., & Shinoda-Mettler, A. (2006). A competency-based approach to public health nursing performance appraisal. *Public Health Nursing, 23*(2), 115-138 124p.

NACNS. (2010). *Clinical Nurse Specialist Core Competencies*. Retrieved from

Nieminen, A.-L., Mannevaara, B., & Fagerström, L. (2011). Advanced practice nurses' scope of practice: a qualitative study of advanced clinical competencies. *Scandinavian Journal of Caring Sciences, 25*(4), 661-670 610p. doi:10.1111/j.1471-6712.2011.00876.x

NONPF. (2014). Nurse Practitioner Core competencies. Retrieved from http://c.ymcdn.com/sites/www.nonpf.org/resource/resmgr/competencies/npcorecompetenciesfinal2012.pdf
